# Macrophages‐Triggered Sequential Remodeling of Endothelium‐Interstitial Matrix to Form Pre‐Metastatic Niche in Microfluidic Tumor Microenvironment

**DOI:** 10.1002/advs.201900195

**Published:** 2019-04-10

**Authors:** Hyunho Kim, Hyewon Chung, Jaehoon Kim, Dong‐Hee Choi, Yoojin Shin, Yong Guk Kang, Beop‐Min Kim, Sang‐Uk Seo, Seok Chung, Seung Hyeok Seok

**Affiliations:** ^1^ School of Mechanical Engineering College of Engineering Korea University Seoul 02841 Republic of Korea; ^2^ Department of Microbiology and Immunology Institute of Endemic Disease College of Medicine Seoul National University Seoul 03080 Republic of Korea; ^3^ Department of Mechanical Engineering Massachusetts Institute of Technology Cambridge MA 02139 USA; ^4^ Department of Bio‐Convergence Engineering College of Health Science Korea University Seoul 02841 Republic of Korea; ^5^ Department of Biomedical Sciences College of Medicine Seoul National University Seoul 03080 Republic of Korea; ^6^ KU‐KIST Graduate School of Converging Science and Technology Korea University Seoul 02841 Republic of Korea

**Keywords:** 3D microfluidic tumor microenvironment, macrophages, metastasis, monocytes, pre‐metastatic niches

## Abstract

The primed microenvironment of future metastatic sites, called the pre‐metastatic niche, is a prerequisite for overt metastasis. However, a mechanistic understanding of the contributions of recruited cells to the niche is hindered by complex in vivo systems. Herein, a microfluidic platform that incorporates endothelial cells and extracellular matrix (ECM) scaffolds is developed, and the distinct role of recruited monocytes and macrophages in establishing pre‐metastatic niches is delineated. It is observed that monocyte‐derived matrix metalloproteinase 9 facilitates cancer cell extravasation through destruction of endothelial tight junctions. Furthermore, subsequent cancer cell invasiveness is significantly enhanced. Close examination of ECM structures reveals that cancer cells move within characteristic “microtracks” generated by macrophages, suggesting that macrophages could serve as a compensatory mechanism for the reduced migratory capacity of cancer cells. Thus, the first evidence of monocyte/macrophage‐induced remodeling is shown, and these findings will open up new horizons for improving characterization of the pre‐metastatic niche and corresponding immunotherapies.

## 1 Introduction

When shed into the bloodstream, disseminating cancer cells must complete a sequential metastatic cascade to eventually give rise to overt metastasis, which is a rare event despite millions of cancer cells shed from primary tumor every day.^1^, ^2^ The key determinant of this fate between death and survival could be a permissive microenvironment, which could enhance cancer cell homing and colonization.^3^, ^4^ Importantly, establishment of the microenvironment at a future metastatic site, called a pre‐metastatic niche, even precedes cancer cell access.^5^, ^6^, ^7^ Indeed, it is well documented that vascular endothelial growth factor receptor 1 (VEGFR1)‐positive bone marrow derived cells (BMDCs) are recruited to pre‐metastatic sites.^8^ Myeloid cells, including myeloid‐derived suppressor cells (MDSCs), CD11b^+^ myeloid cells, and tumor‐associated macrophages are recognized as major initiators in the pre‐metastatic niche.^9^, ^10^, ^11^, ^12^ However, subsequent aspects of the cooperative interaction between these recruited myeloid cells and cancer cells to initiate the metastatic cascade have not yet been elucidated.

As one of the highly abundant components in the tumor microenvironment, monocytes and their differentiated tumor associated macrophages (TAMs) orchestrate tumor metastasis through modulation toward a cancer cell‐favorable microenvironment.^13^, ^14^, ^15^, ^16^ Furthermore, the implication of TAMs on the pre‐metastatic niche reconstitution has been demonstrated by the observation that recruitment of monocytes and macrophages is required for subsequent cancer cell survival^17^ and other similar in vivo studies.^9^, ^10^, ^18^ Recently, in addition to recruited populations, Sharma and colleagues^18^ highlighted that pulmonary alveolar macrophages are also recruited by the primary tumor to foster an immunosuppressive microenvironment in the pre‐metastatic organ. Thus, macrophage‐mediated formation of the pre‐metastatic niche appears to be very necessary for successful metastasis. To date, for further characterization of TAMs in the pre‐metastatic niche as a plausible target for future immunotherapy, substantial data have found the critical molecular component, particularly supporting TAM recruitment in the niche, using an unbiased approach, such as genomic and proteomic profiling.^6^, ^11^, ^19^, ^20^ Nevertheless, in vivo complexity of the pre‐metastatic niche characterized by the interaction among a number of myeloid cells, secreted factors, and/or cell‐to‐cell interactions, makes it still challenging to dissect the distinct contribution of TAMs in the formation of the pre‐metastatic niche.

Hence, we proposed an in vitro 3D microfluidic model that allows direct observation of interaction between macrophages and cancer cells as well as strict regulation of cellular and molecular factors in the niche. Using the model, we investigated whether recruitment of monocytes and subsequent macrophages, prior to cancer cells, created a supportive microenvironment that facilitated cancer cell extravasation and invasion into neighbors. The results presented here extended beyond the limits of existing approaches and ultimately expand therapeutic strategies to effectively inhibit metastasis from a new angle.

## 2 Results

### 2.1 Creation of 3D Pre‐Metastatic Niche in a Microfluidic Model

**Figure 1:**
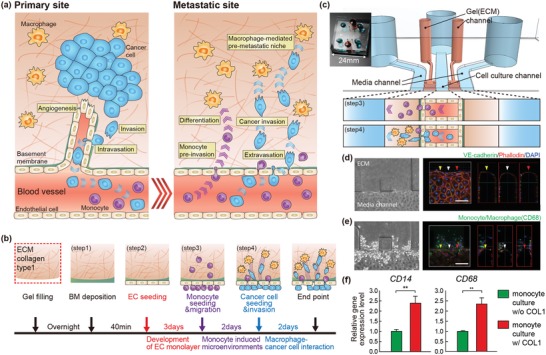
Creation of physiologically relevant pre‐metastatic niche induced by recruited inflammatory monocytes and subsequent macrophages in a microfluidic model. a) Schematic overview of the microenvironment incorporating recruited inflammatory monocytes, subsequent macrophages and following cancer cells in pre‐metastatic niche. b) Procedure of microfluidic device to reconstitute the physiologically relevant 3D microenvironment induced by recruited inflammatory monocytes and macrophages to promote cancer cell invasion in a microfluidic model (step 1–4). c) Photograph and schematic of the microfluidic device. (Left) Photograph of the microfluidic device incorporating hydrogel and colored liquids in the side channels (blue) and central channel (orange). (Right) Schematic images showing invasion of monocytes (step 3) and cancer cells (step 4) into the hydrogel. d) The endothelial network in the 3D model is confirmed by endothelial adherens junction molecule VE‐cadherin (green) (Scale bar = 150 µm). e) Representative confocal fluorescence images showing invasion of monocytes (CD68, green) in established 3D model. d,e) Yellow, white, and red arrow heads indicate each position of cross‐section view (right panels) (Scale bar = 150 µm). F‐actin (red) and DAPI (nucleus, blue). f) mRNA expression levels of markers for macrophage differentiation, CD14 and CD68 in monocytes under 3D culture embedded in type 1 collagen hydrogel. *n* = 3 independent experiments. Data represent mean ± S.E.M. **, *p* < 0.01 by Student's *t*‐test.

To investigate how monocytes and macrophages affect cancer cell extravasation and invasion, a new in vitro system to create physiologically relevant microenvironments is essential (**Figure** **1**). To this end, we have developed an in vitro platform that can mimic the niche's physiological microenvironment using a microfluidic chip. For monocytes and macrophages as well as cancer cells to invade new metastatic organs during extravasation, there are two major barriers, EC monolayer and basement membrane in the ECM (Figure 1a). Successful passage of these barriers might be a rate‐limiting step for extravasation and invasion into neighbors. We, therefore, have integrated the EC monolayer with the basement membrane for extravasating monocytes and sequentially following cancer cells in the microfluidic model. As shown in Figure 1b,c, four steps were required to reconstitute the complicated pre‐metastatic niche in a microfluidic channel. The first step was to form a collagen type 1 hydrogel, a main component of the ECM, to create an environment for cell growth and invasion. Second, we created a constructive EC monolayer on a locally reconstituted thin basement membrane as previously described.^21^, ^22^ Functionality of the EC monolayer was confirmed by expression of endothelial adherens junction, vascular endothelial (VE)‐cadherin (Figure 1d). Monocytic THP‐1 cells were introduced into the microchannel, attached on the EC monolayer, and then extravasated into neighboring ECM for 2 d (Figure 1e). Monocytes were seeded on the EC monolayer to imitate the early events of forming the pre‐metastatic niche, and bone marrow‐derived monocytes adhered on the vascular endothelium. This process allowed us to focus on the unique contribution of monocytes and subsequent macrophages to the pre‐metastatic niche formation after the sequential processes of recruitment, arrest, and adhesion. Consistent with previous result,^23^, ^24^ we found, while migrated within the collagen scaffold, the monocytes differentiated to macrophages as confirmed by increased expression of markers associated with macrophage differentiation such as CD14, CD68, and CD11b^25^, ^26^ and even toward to M2 phenotype with high CD206 expression rather than M1 phenotype (Figure 1f and Figure S1, Supporting Information). Finally, GFP‐tagged MDA‐MB‐231 cells, metastatic breast cancer line, were introduced in the same manner as monocytes. Their 3D and collective responses by interacting with the modified EC monolayer and remodeled ECM by pre‐invaded monocytes and macrophages were monitored for the next 2 d.

In the microfluidic channels, the pre‐invaded monocytes and macrophages affected both the vascular endothelium and ECM, and they altered the microenvironment favoring extravasation and invasion of cancer cells (Figure 1b). For the first time, the microfluidic system incorporated with major niche components can successfully simulate the extracellular microenvironment used by monocytes and macrophages in the in vivo pre‐metastatic niche, enabling deeper studies on cancer cell interactions with monocytes and macrophages that lead to metastasis.

### 2.2 Increased Vascular Permeability through Destruction of Endothelial Tight Junctions by Monocytes

**Figure 2:**
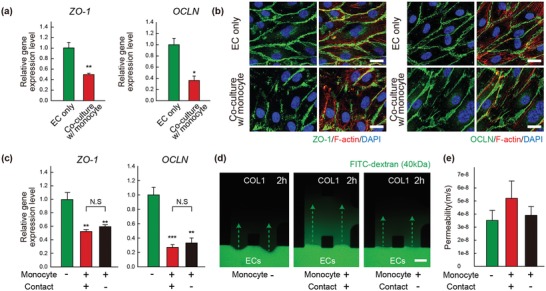
Monocytes‐induced destruction of endothelial tight junctions and increased vascular permeability. a) mRNA expression levels of ZO‐1 and occludin (OCLN) in endothelial cell only or in a coculture with monocytes for 30 min. b) Representative confocal immunofluorescence images of endothelial cells stained for endothelial tight junctions ZO‐1 (green) and OCLN (green), F‐actin (red) and DAPI (nucleus, blue) after coculturing with or without monocytes for 24 h in microfluidic model (Scale bar = 25 µm). c) mRNA expression levels of ZO‐1 and OCLN in endothelial cell with/without monocytes. Endothelial cells in direct contact with monocytes were compared to those with noncontacting monocytes after 30 min of coculture. d,e) Representative fluorescence images and quantification showing diffusion of FITC‐dextran (40 kDa) (green) across the endothelial cell monolayer for 2 h (Scale bar = 150 µm). Data represent mean ± S.E.M. *n* = 3 independent experiments. *, *p* < 0.05, **, *p* < 0.01, ***, *p* < 0.001 by Student's *t*‐test. N.S., nonsignificant.

Destabilization of the endothelium has been observed in the pre‐metastatic niche and is directly related to cancer extravasation and metastasis.^27^, ^28^, ^29^ To investigate the role of monocytes on destabilization, destruction of the vascular endothelial barrier and increased permeability by monocytes were analyzed. We hypothesized that monocytes regulated tight junctions and increased permeability, considering the well‐characterized role of tight junctions to stabilize vessel integrity and their dysregulation in the tumor microenvironment.^30^, ^31^, ^32^ When cocultured with monocytes, endothelial cells (ECs) presented a remarkable decrease in both mRNA levels of two tight junction proteins, ZO‐1 and occludin (OCLN) (**Figure** **2**a). Immunofluorescent‐stained tight junction protein signals were also reduced in the chip microvasculature with monocytes (Figure 2b).

Next, the reduction of ZO‐1 and OCLN was examined to determine whether the cause was due to direct contact or secreted factors. As shown in Figure 2c, the reduced expression was unchanged in endothelial cells cocultured with, but not contacting, monocytes (noncontact). It strongly suggested the involvement of soluble factors from monocytes rather than contact‐dependent signals. With the reduced ZO‐1 and OCLN expression, increased vascular permeability was observed in the microfluidic system (Figure 2d,e). They suggested that monocyte‐secreted soluble factors were a potent regulator of tight junction protein ZO‐1 and OCLN to destroy the integrity of vascular barrier against metastasis.

### 2.3 Endothelial Barrier Disruption by Monocyte‐Derived Matrix Metalloproteinase 9 (MMP9) to Promote Cancer Cell Extravasation

**Figure 3:**
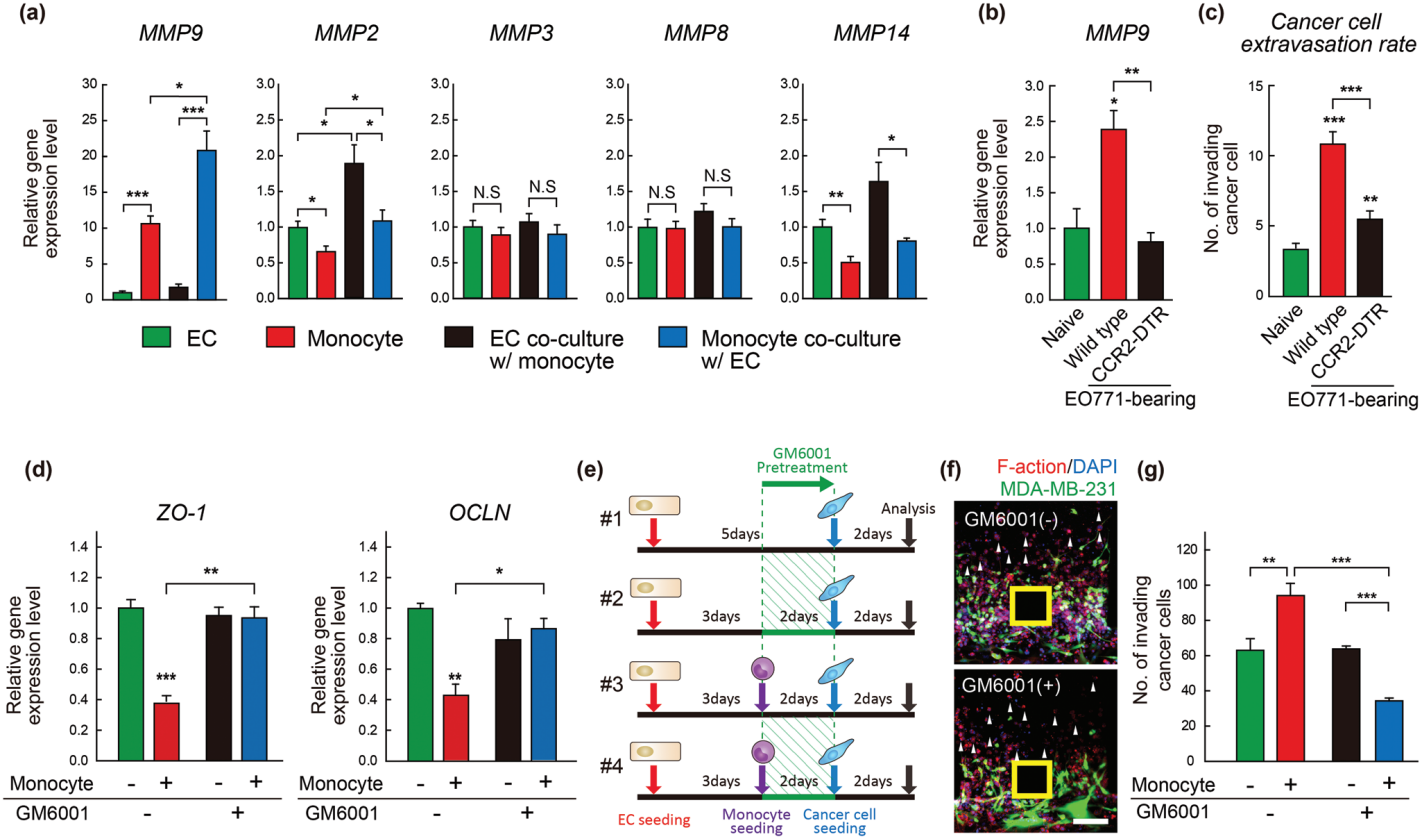
Monocyte‐derived MMP9 promotes cancer cell extravasation. a) mRNA expression levels of various MMP subtypes (MMP2, 3, 8, 9, and 14) in endothelial cells, monocytes, cocultured endothelial cells, and cocultured monocytes. *n* = 3 independent experiments. b,c) Wild‐type mice and CCR2‐DTR mice were orthotopically injected EO771 mouse breast cancer cells following intraperitoneal administration of diphtheria toxin (DT) three times a week to deplete CCR2‐expressing inflammatory monocytes, thereby macrophages. b) 14 d after the EO771 injection, MMP9 mRNA in the pre‐metastatic lung was analyzed in naïve (tumor‐free), EO771‐bearing wild‐type mice, and CCR2‐DTR mice treated with DT. c) Cancer cell extravasation to the lungs of naïve, EO771‐bearing wild‐type, and CCR2‐DTR mice at day 14 post‐tumor injection, after 24 h after intravenous injection of fluorescently labeled EO771 cells, measured by the number of cancer cell in microscopic field. *n* = 5/group. d) mRNA expression levels of ZO‐1 and OCLN in endothelial cells only or cocultured with monocytes upon MMP9 inhibitor, GM6001, treatment. *n* = 3 independent experiments. e) Illustration of the experiment for MMP9 inhibition using GM6001 in microfluidic model. f) Representative confocal fluorescence images showing invasion of GFP‐tagged MDA‐MB‐231 cells in a presence of pre‐invaded monocytes with/without GM6001 treatment for 2 d. The images are representative of >10 samples. Arrow heads point to the pre‐invaded monocytes (Scale bar = 150 µm). F‐actin (red) and DAPI (nucleus, blue). g) Quantification of extravasation of MDA‐MB‐231 cell as defined by the number of GFP‐tagged cells under the conditions of with/without pre‐invaded monocytes and with/without GM6001 treatment in microfluidic model. *n* = 9–11. Data represent mean ± S.E.M. *, *p* < 0.05, **, *p* < 0.01, ***, *p* < 0.001 by Student's *t*‐test.

MMP9 secreted by monocytes is known to decrease vascular integrity by local disappearance of the tight junction protein OCN in brain endothelial cells.^33^ We thus hypothesized that MMP9 could be a culprit in the observed monocyte‐mediated destruction of the vascular integrity. Expression levels of various MMP subtypes (MMP2, MMP3, MMP8, MMP9, and MMP14) in endothelial cells and monocytes were first measured in 2D culture systems. The expression of MMP3 and MMP8 was similar and that of MMP2 and MMP14, known to be associated with angiogenesis,^34^, ^35^ is slightly lower (1.5‐fold and 2‐fold, respectively) in monocytes, whereas MMP9 is remarkably highly expressed in monocytes (more than 10‐fold) in comparison with endothelial cells (**Figure** **3**a). The difference in MMP9 expression became even more apparent (13‐fold) upon coculture with other cells but not in other MMP subtypes. MMP9 secretion by monocytes in the pre‐metastatic niche was also confirmed in vivo with CCR2‐DTR mice, in which CCR2^+^ inflammatory monocytes were selectively ablated upon diphtheria toxin (DT) treatment.^36^ Macrophages were depleted in the pre‐metastatic lung of EO771 breast tumor‐bearing CCR2‐DTR mice by DT treatment to block CCL2‐CCR2‐dependent recruitment of inflammatory monocytes.^37^ Compared with naïve mice, the MMP9 expression level was significantly elevated in pre‐metastatic tumor‐bearing mice as reported.^7^, ^8^, ^9^, ^38^ Interestingly, reduced expression in the lungs of CCR2‐DTR mice treated with DT was measured, identifying monocytes as a major source of MMP9 in the pre‐metastatic niche (Figure 3b).

Consistently, monocyte‐dependent cancer cell extravasation was also observed (Figure 3c). The monocyte‐derived MMP9 lowered expression level of tight junction proteins, ZO‐1 and OCLN, in endothelial cells and seemed to destroy the endothelial barrier, thereby increasing vascular permeability. GM6001 (MMP9 inhibitor) treatment restored the expression level of tight junction proteins in endothelial cells, regardless of monocytes coculture (Figure 3d). In sequential triculture of endothelial cells, monocytes, and cancer cells, cancer cell invasion was not affected by GM6001 treatment in endothelial cells without monocytes (Figure 3e–g). Only GM6001 treatment in monocytes could effectively abolish the extravasation and subsequent invasion of cancer cells. In experiments, MMP9 function in monocytes was inhibited for only 2 d while monocytes invaded (Figure 3e). Before cancer cell introduction, thorough washing occurred to remove the direct effect of GM6001 on cancer cells. Experiments of GM6001 treatment only in endothelial cells could support negligible contributions of endothelial cell‐derived MMP9^9^ in cancer cell extravasation (Figure 3g). Unlike the similar expression of tight junction proteins between the conditions Monocyte/GM6001 (−/−) and Monocyte/GM6001 (+/+) (Figure 3d), it was notable that in the presence of GM6001‐treated monocytes (+/+), cancer cell extravasation even more significantly reduced than condition monocyte/GM6001 (−/−) (Figure 3g). It might be explained by the fact that despite blockade of MMP9 function, monocytes could invade the matrix using the MMPs‐independent amoeboid migration mode,^39^ as confirmed by no difference in monocyte migration between the conditions Monocyte/GM6001 (+/−) and (+/+) (Figure 3f). The pre‐invaded GM6001‐treated monocytes formed cell–cell contact‐based junction in initial phase of invasion within the matrix (<150 µm), and then worked as a barrier for subsequent cancer cells to invade and further hampering cancer cell extravasation in addition to impaired MMP9 function.

These observations clearly illustrated that monocyte‐derived MMP9 facilitated cancer cell extravasation by regulating tight junction proteins, ZO‐1 and OCLN.

### 2.4 Enhanced Cancer Cell Invasiveness by Pre‐Invaded Macrophages

**Figure 4:**
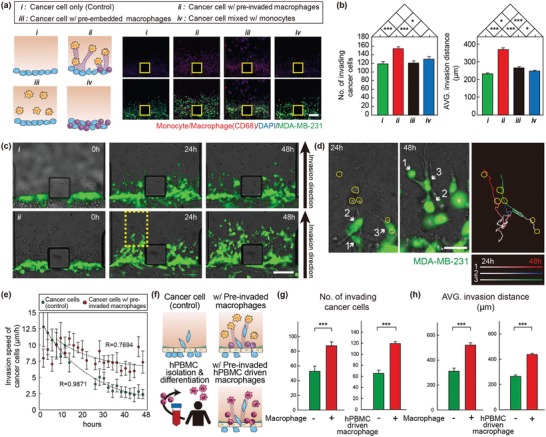
Cancer cell invasiveness within the ECM matrix is sustained by macrophages. a) Illustration of the experiment for comparing cancer cell invasiveness under four coculture conditions: (i) cancer cell only, (ii) with pre‐invaded macrophages into ECM, (iii) with pre‐embedded macrophages into ECM, and (iv) monocytes mixed with cancer cells (Scale bar = 150 µm). Monocytes/macrophages (CD68, green), MDA‐MB‐231 cells (green), and DAPI (nucleus, blue). b) Quantification of MDA‐MB‐231 cells invasion as defined by the number of GFP‐tagged cancer cells (left panel) and average invasion distance (right panel) under the four respective conditions. c) Time‐lapse images of GFP‐tagged MDA‐MB‐231 cells in the absence (i, upper panels) or presence (ii, lower panels) of pre‐invaded macrophages within the matrix (0, 24, 48 h after cancer cell seeding) (Scale bar = 150 µm). d) Time‐lapse images of GFP‐tagged MDA‐MB‐231 cells (green) in the presence of pre‐invaded macrophages from the yellow dotted line in (c). Yellow circles indicate pre‐invaded macrophages within the matrix before cancer cell invasion. Rightmost panel shows tracking data for a subset of cells. Tracked cancer cells labeled as cell 1, 2, or 3 for comparison with tracks. The images are representative of 15 samples (Scale bar = 50 µm). e) Speed of MDA‐MB‐231 invading through collagenous ECM in the absence or presence of pre‐invaded macrophages measured every 2 h. *n* = 15. f) Illustration of the experiment using THP‐1 and human PBMC‐driven macrophage. g,h) Quantification of MDA‐MB‐231 cells invasion as defined by the number of GFP‐tagged cancer cells and average invasion distance in the presence of THP‐differentiated macrophage (left panel) and human PBMC‐driven macrophage (right panel). *n* = 8–9. Data represent mean ± S.E.M. *n* = 3 independent experiments. *, *p* < 0.05, ***, *p* < 0.001 by Student's *t*‐test.

Following extravasation, we next studied the further role of pre‐invaded monocytes to facilitate subsequent cancer cell invasion within the ECM matrix. Cancer cell invasiveness under four coculture conditions was compared: (a) without monocytes/macrophages, (b) with pre‐invaded macrophages into ECM, (c) with pre‐embedded macrophages into ECM, and (d) monocytes mixed with cancer cells (**Figure** **4**a).

In the presence of pre‐invaded macrophages, as confirmed by increased CD14, CD68, and CD11b expression (Figure 1f and Figure S1, Supporting Information), cancer cell invasiveness determined by the number of invaded cells and invasion distance within the ECM significantly increased, suggesting that during invasion into the ECM, macrophages formed a microenvironment favorable for cancer cell invasion (Figure 4b). The time course during invasion within the matrix was carefully analyzed with GFP‐tagged cancer cells in the microfluidic coculture model. Time‐lapse images showed that the cancer cells tended to move toward pre‐invaded macrophages (Figure 4c,d). Quantified cancer cell invasion speed (measured at 2 h intervals) showed that cancer cells actively invaded into ECM, and after 12 h, they started to lose their invasiveness. However, when invading into the macrophages‐invaded ECM, cancer cells maintained their invasiveness as long as 48 h (Figure 4e).

These results suggested the existence of a physical or chemical guidance signal, which was formed during macrophage migration. The guidance seemed to work as a “slippery slope” to direct persistent cancer cell invasion into the ECM. It was proven by the increased cancer cell penetration through the EC monolayer and their invasion distance after extravasation, respectively (Figure 4f–h). Similar enhancement of cancer cell extravasation and invasion was noticed when cultured with macrophages differentiated from human peripheral blood mononuclear cells (hPBMC‐driven macrophages) (Figure 4f–h).

### 2.5 Macrophage‐Mediated Dynamic Remodeling of ECM through Establishing Microtracks

**Figure 5:**
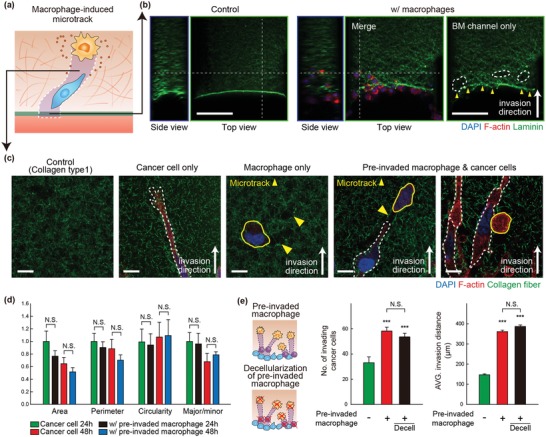
Macrophage‐mediated microtracks generation enhances cancer cell invasion. a) Schematic of macrophage‐mediated microtracks generation within the ECM matrix. b) Representative confocal fluorescence images of laminin (green), F‐actin (red), and DAPI (nucleus, blue) showing basement membrane degradation by pre‐invaded macrophages (right panel) compared to control (left panel). Yellow arrowheads and white circles indicate basement membrane degradation and collagen remodeling within the matrix, respectively (Scale bar = 150 µm). c) Representative confocal fluorescence images of collagen fiber (green), F‐actin (red), and DAPI (nucleus, blue) showing collagen structure without cells, with MDA‐MB‐231 cells (white dotted line), macrophages (yellow line), and cancer cells in the presence of pre‐invaded macrophages, respectively. Yellow arrowheads indicate the microtracks generated by macrophages (Scale bar = 10 µm). d) Quantification of morphological changes in cancer cells as measured by area, perimeter, circularity, and major/minor ratio in the absence or presence of pre‐invaded macrophages at 24 and 48 h after cancer cell seeding in microfluidic model. e) Illustration of the experiment for decellularization of pre‐invaded macrophages (left panel) and quantification of MDA‐MB‐231 cells invasion as defined by the number of GFP‐tagged cancer cells (middle panel) and average invasion distance (right panel) in the presence of pre‐invaded macrophage with/without decellularization process. *n* = 10–11. Data represent mean ± S.E.M. *n* = 5 independent experiments. ***, *p* < 0.001 by Student's *t*‐test. N.S., nonsignificant.

Mechanism of the “slippery slope” for monocytes and subsequent macrophages was assessed with the EC monolayer cultured on the basement membrane over an interstitial matrix in the microfluidic platform (**Figure** **5**a). Next, to find the direct effect of macrophages on the remodeling of collagen matrix after passing through endothelium (extravasation), we observed the interactions among pre‐invaded macrophages, cancer cells, and collagen fibers in microfluidic devices. We used the same experimental conditions but without endothelial cells. Macrophages first breached the basement membrane as confirmed by laminin degradation (Figure 5b), followed by the remodeled interstitial matrix. The macrophage‐mediated remodeling was visualized by immunofluorescent staining of fibrillar collagen type I, a predominant component of the matrix. Figure 5c and Figure S2 (Supporting Information) showed empty space (microtracks) within the matrix, where collagen fibers were realigned and degraded by invaded macrophages. The second harmonic generation (SHG) imaging microscopy of collagenous matrix in the presence of macrophages also demonstrated the formation of invadopodia during macrophage migration and thereby realigned collagen fiber (Figure S3, Supporting Information), directly proving modification of collagen by macrophages in their migration. Importantly, when cancer cells were seeded subsequently, the cancer cells moved within the tracks formed behind the pre‐invaded macrophages, and even cancer cells in direct contact with macrophages were observed, which could also play a role in facilitating cancer cell invasion within the matrix (Figure 5c).^39^ However, individual cancer cell morphology was not changed by macrophage‐formed microtracks. When morphological indices, including area, perimeter, circularity, and aspect ratio, were measured on 3D invading single cancer cells in the matrix, no significant difference was found in the quantified indices of invading cancer cells with or without pre‐invaded macrophages (Figure 5d). Rather, analysis of cancer cell 3D morphology proved a decrease in intrinsic migratory capacity of cancer cells into the ECM. In 24 h, area, perimeter, and aspect ratio of invading cancer cells decreased, while circulatory increased, indicating that the cancer cell morphology became less bipolar over time and there was reduced migratory capacity in the 3D invasion. It indirectly suggested that the overall increase in invasiveness of cancer cells was not due to the increased mobility of cancer cells.

We next investigated whether macrophage‐driven microtracks generation is sufficient for guidance of subsequently invading cancer cells independent of macrophage‐derived factors. Before cancer cell seeding, pre‐invaded macrophages and their secreted factors could be removed by deionized water (DW) treatment for 2 h after their invasion. Cancer cells were then seeded on the macrophage‐invaded EC monolayer (on basement membrane over interstitial matrix) with or without the DW treatment (Figure 5e). Macrophages exposed to DW were confirmed to be dead by trypan‐blue staining (Figure S4, Supporting Information). Surprisingly, no difference in cancer cell invasiveness was observed in the absence of pre‐invaded macrophages and their secreted factors, suggesting that the factors produced by macrophages were unlikely to be responsible for the increased cancer cell invasion (Figure 5e). The macrophage‐driven remodeling of the basement membrane‐interstitial matrix continuum was verified to work as a slippery slope, physically directing persistent 3D invasion of cancer cells notwithstanding decreased intrinsic migratory capacity.

## 3 Discussion

New evidence may hold important clues for the possibility of targeting macrophages at pre‐metastatic niche sites to overcome current limitations in conventional anticancer therapies.^3^, ^14^, ^17^, ^40^ However, confronting the methodological difficulties of observing direct crosstalk between macrophages and cancer cells in the niche, recent studies have failed to fully uncover the important aspects of macrophages that enhance cancer cell extravasation and subsequent invasion in in vivo pre‐metastatic niches. These unmet needs were met by a novel 3D microfluidic system to investigate macrophage dynamics in a reconstituted 3D pre‐metastatic niche. It has been shown that monocytes increased vessel permeability through destruction of the endothelial barrier and thus enhanced the extravasation rate of cancer cells. This is consistent with previous reports on the decreased vascular permeability upon monocyte depletion with clodronate liposomes^41^ and the primary tumor‐induced hyperpermeability in pre‐metastatic organs in vivo.^28^, ^29^

MMP9 was identified as a key factor in monocyte‐mediated extravasation by breaking the vessel barrier, as confirmed by significantly reduced extravasation rates of cancer cells via inhibited MMP9 function in monocytes. Of note, MMP9 identified in our work has also been described as a culprit of organ‐specific metastases and is mainly expressed by infiltrated macrophages in pre‐metastatic organ.^9^ The previous study further supports our observation of significantly reduced MMP9 expression as well as cancer cell extravasation in the pre‐metastatic lung of CCR2‐DTR mice, in which recruitment of inflammatory monocytes via CCL2‐CCR2 axis was blocked, indicating primary tumor‐induced recruited inflammatory monocytes and subsequent macrophages were responsible for MMP9 expression in the pre‐metastatic lung.

It has also been shown that upregulated MMP9 expression in the pre‐metastatic niche is attributed to endothelial cells, besides macrophages.^9^ However, our analysis of treatment using the MMP inhibitor in endothelial cells resulted in no disadvantages in extravasation rates of cancer cells. In contrast, treatment of monocytes led to remarkable attenuation. Our data, therefore, suggest the significance of increased MMP9 expression in monocytes and subsequent macrophages, rather than endothelial cells, to open the endothelial barrier in pre‐metastatic niches. Further, it suggests that the unidentified roles of endothelial cell‐derived MMP9 in pre‐metastatic niches, other than increasing vascular permeability shown in monocytes, remain to be elucidated.

We further demonstrated that macrophage‐dependent physical matrix remodeling is dominant over cancer‐cell intrinsic properties in persistent cancer cell invasiveness. When macrophages actively invaded within the 3D interstitial matrix, they generated tunnel‐like structures, microtracks, which could enable persistent invasion of subsequent cancer cells. These observations dramatically showed pre‐metastatic matrix formation, partly in line with previous findings that macrophages formed microtracks within the Matrigel matrix.^42^ However, we incorporated collagen type I scaffolds in our microfluidic platform to mimic the interstitial matrix more closely to the in vivo tumor microenvironment. Although it has been reported that pure collagen has poor mechanical properties,^43^, ^44^ 2.0 mg mL^−1^ of type I collagen hydrogel, used in our study, showed enhanced mechanical properties with stiffness about 42.8 kPa.^45^ Thus, we could mimic extracellular matrix in metastatic breast cancer tissue with stiffness value of about 48 kPa by type I collagen hydrogel.^46^ In the microfluidic platform, cancer cells could exhibit two invasion modes: macrophage‐dependent or independent, which were quite different from absolute dependency of cancer cells on macrophages to invade as shown in a previous study.^42^ Given that aggressive cancer cells with sufficient capability to metastasize to other organs could induce pre‐metastatic niches,^3^, ^5^, ^6^ the 3D basement membrane‐interstitial matrix continuum incorporated with type I collagen would be far more appropriate for physiologically relevant studies related to the pre‐metastatic niche rather than the Matrigel matrix. The characteristic structure of macrophages‐driven microtracks shown as empty space within the collagen hydrogel suggests that pre‐invaded macrophages undergo a proteolytic migration mode that leads to form microtracks. A recent study also found that cells could adopt protease‐independent migration through plastic matrix using mechanically widening and lengthening protrusion to open up permanent channel to migrate through.^47^ Taken together, it is thought that a combination of protease and force‐mediated remodeling of collagenous matrix by pre‐invaded macrophages is attributable to microtracks through which cancer cells can subsequently invade.^47^, ^48^

Fibroblasts have also been reported to form microtracks with similar structure characterized by holes in the matrix behind the invaded cells, thereby leading to collective invasion of cancer cells with epithelial characteristics.^49^ However, according to the report, there is little effect of fibroblast‐driven microtracks on invasion of cancer cells with mesenchymal phenotypes, which are hallmarks of metastatic cancer cells. Similarly, we also observed that MDA‐MB‐231 cells, one of the aggressive breast cancer cells with mesenchymal phenotypes, invaded well regardless of the presence of pre‐invaded monocytes in a short distance (<150 µm, <24 h) (Figure 4). Strikingly, real‐time observation of invading cancer cells found that macrophage‐driven microtracks were likely to be important for successful cancer cells invasion deep into the matrix (>150 µm, >24 h), where their migratory capacity is reduced.

Thus, we uncovered that reduced cell‐intrinsic aggressiveness of cancer cells, presumably at the invasive front, could be compensated by preformed microtracks within the ECM, which could not be recapitulated in vivo. Although prior studies have implicated macrophages or fibroblasts in the generation of microtracks in the 3D tumor microenvironment, this study, to our knowledge, is the first to report that cancer cells, even highly aggressive cancer cells with mesenchymal characteristics, critically depend on compensation mechanisms via microtracks formed by pre‐invaded macrophages. Furthermore, the equally enhanced cancer cell invasion, despite macrophages‐decellularized matrix, reveals that pre‐invaded macrophages could modify composition and structure of the matrix to establish hospitable geometry of stromal ECM which is sufficient for cancer cells to sustain invasiveness with little requirement for soluble factors. Given a well‐established critical role of macrophages in the deposition, cross‐linking and linearization of collagen,^13^ besides microtracks generation, macrophages‐triggered deformation of the matrix could also increase local matrix stiffness or establish plasticity in this context,^47^, ^48^ and thereby provide cooperative mechanisms of enhanced cancer cell invasiveness in the decellularized matrix.^47^, ^50^, ^51^ These findings strongly suggest that dissemination of cancer cells could be decided, in part, by macrophage‐mediated pre‐metastatic niches and in this regard, it is likely that conventional therapeutic approaches regulating cancer cells only, with little consideration for macrophages in the pre‐metastatic niche, possibly lead to the limited effect on metastatic inhibition.

**Figure 6:**
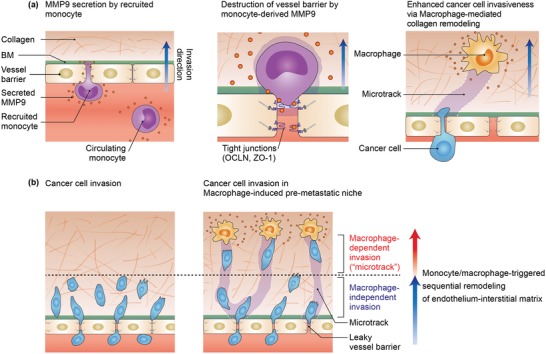
A proposed mechanism of monocyte/macrophage‐mediated formation of pre‐metastatic niche to facilitate metastatic cascades. a) The establishment of pre‐metastatic niche through monocyte/macrophage‐mediated sequential remodeling of endothelium‐interstitial matrix continuum. Inflammatory monocytes are recruited by primary tumor‐derived factors in pre‐metastatic organ and secret MMP9. Monocytes‐derived MMP9 destructs the endothelial tight junction protein occludin (OCLN) and ZO‐1 to enhance cancer cell extravasation. Upon migration within the ECM matrix, macrophages generate microtracks which are sufficient for subsequent cancer cells to sustain invasiveness within the matrix. b) Ultimately, monocyte/macrophage‐mediated favorable microenvironment for cancer cell results in increased cancer cell extravasation as well as further invasion, leading to a slippery slope toward metastasis.

In conclusion, we took the first step in providing the direct evidence for a critical contribution of monocytes and macrophages to establish permissive microenvironments. Our findings identified unappreciated roles of monocyte‐derived MMP9 to create a permeable EC monolayer, and further revealed that macrophages generated microtracks to allow extravasated cancer cells to sustain high invasiveness regardless of intrinsic metastatic potential (summarized in **Figure** **6**). In addition to well‐described recruitment of monocytes and macrophages in the pre‐metastatic niche, our findings therefore shed light on the pivotal role of macrophages as pioneers leading the slippery slope from opening the endothelial barrier to assisting subsequent invasion within the matrix. Moreover, our 3D microfluidic system can be presented as a platform for studying the crosstalk between macrophages and cancer cells in the pre‐metastatic niche as well as screening associated anticancer therapies.

## 4 Experimental Section

*Preparation of Microfluidic Chip*: Design of the microfluidic chip and manufacturing method were reported in previous studies.^22^ Briefly, SU‐8 photoresist 120–130 µm thick was first photolithographed and patterned on a silicon wafer. Polydimethylsiloxane mixture (PDMS, SYLGARD 184 Silicone Elastomer Kit, Dow Corning) was prepared by mixing the curing agent and PDMS prepolymer in a weight ratio of 1:10. The PDMS mixture was poured onto the patterned wafer. Subsequently, the PDMS mixture was degassed in a vacuum chamber for 15 min to remove bubbles. The PDMS mixture was baked in a drying oven at 80 °C for 4 h. The cured PDMS mold was detached, trimmed, and punched by a biopsy punch and blunt needle. The PDMS mold was autoclaved at 120 °C for 30 min. The sterilized PDMS mold (upper part) and glass coverslip (bottom part) were bonded after oxygen plasma treatment (FEMTO science). Immediately, the bonded microchips were filled with poly‐d‐lysine solution (PDL, 1 mg mL^−1^, Sigma Aldrich), and incubated in a 37 °C incubator for 2 h. After incubation, the microchip was washed twice with deionized water. The microchip was then dried in an oven at 80 °C for over 1 d.

*Preparation of Type 1 Collagen Hydrogel*: The collagen solution was prepared by mixing the rat‐tail collagen type 1 solution (BD), phosphate buffered saline (PBS, 10×, GIBCO), distilled water, and sodium hydroxide (NaOH, 0.1 n). The final concentration and pH of the collagen gel mixture were adjusted at 2.0 mg mL^−1^ and pH 7.0–7.4, respectively. The collagen solution was injected into the gel channel and incubated in a CO_2_ incubator at 37 °C for 30 min at high humidity to avoid drying during gelation. After gelation, basal or growth medium was filled in the media channels.

*Basement Membrane Deposition*: To create a confluent endothelial monolayer, a synthetic basement membrane material, Matrigel (growth factor reduced, Phenol red‐free, BD), was deposited on the collagen hydrogel incorporated in the microfluidic chip.^21^ Matrigel was diluted 1:50 with 4 °C Endothelial Cell Basal Medium‐2 (EBM2, Lonza) (final density of the diluted Matrigel solution was 0.16–0.24 mg mL^−1^ in EBM2). Medium of all reservoirs of the microfluidic chip was aspirated. Further, 50 µL of diluted Matrigel solution was filled into the central medium channel. During handling of the Matrigel, the microfluidic chips were kept at 4 °C. The microfluidic chips were incubated at 37 °C for 40 min, and the channels were rinsed with EBM2.

*Preparation of Cells*: Human dermal microvascular endothelial cells (hMVEC, Lonza) were cultured with Microvascular Endothelial Cell Growth Medium‐2 BulletKit (EGM2‐MV, Lonza). hMVECs were maintained, subcultured, and preserved according to the manufacturer's standard protocol. THP‐1 and green fluorescence protein (GFP) tagged MDA‐MB‐231 cells were cultured in Roswell Park Memorial Institute medium (RPMI) supplemented with 10% fetal bovine serum and 1% penicillin‐streptomycin (PS). The culture medium was refreshed every 2 d and subcultured with 0.25% Trypsin‐EDTA (1×, Phenol Red, Gibco).

Primary macrophages were differentiated from hPBMCs. hPBMCs were isolated from healthy donors (IRB approved C‐1805‐059‐945), and monocytes were isolated using magnetic bead selection (Miltenyi Biotec). Informed consent was obtained from all patients who donated blood. Subsequently, isolated monocytes were cultured in RPMI‐1640 medium containing 10% FBS, 1% PS, and 2 × 10^−3^ m of l‐glutamine supplemented with fresh recombinant human macrophage colony‐stimulating factor (M‐CSF) (50 ng mL^−1^; Miltenyi Biotec) for 7 d to differentiate into mature macrophages.

*Endothelial Cell Monolayer Culture in Microfluidic Chip*: hMVECs that reached 80% confluency in the 75T flask were collected with 0.05% trypsin and neutralized with EGM2‐MV. Briefly, 50 µL of hMVEC suspension prepared at 1.5–2.0 × 10^6^ cells mL^−1^ in EBM was filled in the center channel. After 2 h, the medium in all channels was replaced with 120 µL fresh growth medium and refreshed daily. After 3–5 d of hMVEC culture in the microchannel, the hMVECs formed a confluent EC monolayer on the collagen hydrogel.

*Monocyte Culture in Microfluidic Chip with EC Monolayer*: Monocytes were seeded on the EC monolayer. Monocytes were prepared at 0.5–2.0 × 10^6^ cells mL^−1^ in EGM2‐MV. Briefly, 60 µL of monocyte suspension was added into the microchannel. When the monocytes were uniformly dispersed in the channel, the chip was tilted 90° to allow monocyte seeding on the EC monolayer by gravity. The chips were incubated in a CO_2_ incubator at an inclined position for 2 h. All channels were deliberately replaced with growth medium after the chips were restored to a flat position. Within 2 d, monocytes extravasated through the EC monolayer and migrated into the collagen hydrogel in the ECM channel.

*Cancer Cell Culture on Macrophages Pre‐Invaded ECM in Microfluidic Chip*: MDA‐MB‐231 suspension was prepared at 0.75 × 10^6^ cells mL^−1^ in EGM2‐MV. Briefly, 60 µL of the cell suspension was added into the EC monolayer microchannel. Similar to the monocytes seeding process, the chip was tilted vertically for MDA‐MB‐231 cells to be seeded on the EC monolayer by gravity. After incubation for 2 h in a CO_2_ incubator, the chip was restored to a flat position. All channels were refreshed with EGM2‐MV. Culture medium was replaced with fresh medium daily.

*Decellularization in Microfluidic Chip*: After monocytes migrated into the hydrogel, they were removed without damaging the collagen hydrogel in the ECM channel by replacing the medium of all channels with deionized water. After 2 h of incubation for decellularization, microchannels were rinsed twice with 1× PBS and filled with culture medium. Trypan blue (Gibco, USA) was used to confirm monocyte death. MDA‐MB‐231 cells were seeded and cultured in the same manner as above.

*Real‐Time Quantitative Polymerase Chain Reaction (RT‐PCR)*: RNA was extracted with TRIzol (Invitrogen) according to the manufacturer's instruction. cDNA was synthesized from 1 µg of total RNA, and the mRNA level was determined by quantitative real‐time PCR using the SYBR Green PCR Master Mix (Applied Biosystems). Primers used are listed as follows:

**Table:** 

Primer	Primer sequence (5′→3′)
Zonula occuldens‐1 (ZO‐1)	F: GGG AAC AAC ATA CAG TGA CGC R: CCC CAC TCT GAA AAT GAG GA
Occludin (OCLN)	F: TGG CAA AGT GAA TGA CAA GC R: GCA GGT GCT CTT TTT GAA GG
MMP2	F: GCT GGC TGC CTT AGA ACC TTT C R: GAA CCA TCA CTA TGT GGG CTG AGA
MMP3	F: GAA GAG AAA TTC CAT GGA GCC AGG R: AGA AAT AAA AGA ACC CAA ATT CTT CAA AAA CA
MMP8	F: CCA CTT TCA GAA TGT TGA AGG GAA G R: TCA CGG AGG ACA GGT AGA ATG GA′
MMP9	F: GCA CGA CGT CTT CCA GTA CC R: GCA CTG CAG GAT GTC ATA GGT
MMP14	F: GAG CTC AGG GCA GTG GAT AG R: GGT AGC CCG GTT CTA CCT TC
CD14	F: CAC AGC CTA GAC CTC AGC CAC AAC R: CCA GCC CAG CGA ACG ACA G
CD68	F: CAC CTC CAA GCC CAG ATT CAG AT R: CCT TGG TTT TGT TGG GGT TCA GTA
CD11b	F: CAA GGA AGC CGG AGA GGT CAG A R: CGG AGT CCA GAG CCA GGT CAT AAG
TNF‐alpha	F: CCC AGG GAC CTC TCT CTA ATC A R: GCT TGA GGG TTT GCT ACA ACA TG
CD206	F: CAA GGA AGG TTG GCA TTT GT R: CCT TTC AGT CCT TTG CAA GC
Beta‐actin	F: TCT ACA ATG AGC TGC GTG TG R: ATG GCT GGG GTG TTG AAG

*Immunofluorescent Staining*: Cells in the microfluidic chip were fixed by adding 4% (w/v) paraformaldehyde (in PBS) to each channel of the device and incubating for 15 min at room temperature (RT). Cells were permeabilized with 0.1% Triton‐X100 (Sigma‐Aldrich) in PBS for 5 min, blocked with 20% Block Ace (in PBS) (Dainihon‐Seiyaku, Japan) for 1 h, and washed with PBS. Solutions of rabbit polyclonal anti‐VE‐cadherin (1:100; Abcam, USA), rabbit polyclonal anti‐CD68 (1:100; Abcam) primary antibodies, rabbit polyclonal anti‐Laminin (1:200; Abcam), OCLN (1:100; Thermo Fisher), and ZO‐1 (1:100; Thermo Fisher) were introduced into each device and incubated overnight at 4 °C. After washing with PBS, Alexa Fluor 488‐conjugated goat anti‐rabbit (1:200; Invitrogen, USA) or Alexa Fluor 568‐conjugated goat anti‐rabbit (1:200; Invitrogen) was added to the device and incubated for 2 h. Nuclei and actin filaments were stained with 4′, 6‐diamidino‐2‐phenylindole (DAPI; 1:1000; Invitrogen) and rhodamine phalloidin (1:100; Invitrogen). The stained cells were observed under a fluorescence microscope (Axio Observer D1; Carl Zeiss, Germany) and confocal microscope (LSM‐700; Carl Zeiss).

*Collagen Hydrogel Staining*: After the collagen hydrogel solidified, collagen fibers were stained with Alexa Fluor 488 NHS Ester (Thermo Fisher, USA). The staining solution was diluted to an appropriate concentration (0.5–2 µg mL^−1^ in PBS) and filled into the channels. The microfluidic chip was incubated in the dark for 2 h. After incubation, all channels were washed three times with PBS for 20 min. Subsequently, the basement membrane deposition and cell culture were performed in the same manner.

*Permeability Measurement of EC Monolayer*: Permeability of the EC monolayer was measured using 10 × 10^−6^ m of 40 kDa FITC‐dextran in growth medium. After removing medium from the reservoirs, both sides of the channel were filled with fresh medium, and the EC channel was filled with dextran solution. After 2 h, fluorescent images were obtained. Permeability was evaluated by the fluorescence intensity profile obtained using the ImageJ software. Permeability *P*_D_ was determined as follows

(1) $$P_{D}=\;D\frac{dC}{dx}\frac{1}{\Delta C}$$

where *C* is the concentration of dextran, Δ*C* is the concentration difference across the ECs, d*C*/d*x* is the slope of the concentration profile in collagen scaffold, and *D* is the diffusion coefficient.

*Real‐Time Imaging*: GFP‐tagged MDA‐MB‐231 cells were observed by JuLI Fr (Nanoentek, South Korea). JuLI Fr was placed in an incubator to observe cells in real time. Time‐lapse images were captured every 10 min for 48 h. Live cell tracking was measured using the manual tracking feature of the ImageJ software (version 1.51K).

*Image Processing*: All images were processed, and composites were created using the Adobe Design Premium CS6 software package and ImageJ software. Only contrast and brightness of the original images were adjusted, within linear range.

*SHG Microscopy*: Microtrack of collagen gel was observed by using the Second Harmonic Generation Microscope. Ultrafast laser (Chameleon Ultra II, Coherent) at 810 nm was used as the light source. Second harmonic generation at 405 nm was observed with photon multiplier tube (Hamamatsu) and appropriate bandpass filters. Fluorescence image of cells was also acquired by two‐photon fluorescence process simultaneously. Temperature, humidity, and CO_2_ concentration were all adjusted to maintain the viability of the cells during imaging.

*Tumor Xenograft Model*: All the mice were obtained from Orient Bio (Korea) and maintained under specific pathogen‐free (SPF) conditions according to the Guide for the Care and Use of Laboratory Animals prepared by the Institution of Animal Care and Use Committee of Seoul National University. All of the experiments were approved by the Institute for Animal Care and Use Committee of Seoul National University (Accession No. SNU‐150708‐1‐1). For tumor xenograft models, orthotopic tumor injections were performed by administering 2 × 10^5^ cells in single‐cell suspension of 100 µL of PBS. The EO771 breast carcinoma cell lines were administered into inguinal right fourth mammary fat pads of 7‐ to 8‐week‐old female C57BL/6J mice.

*Statistical Analysis*: All statistical analyses were run using GraphPad Prism 5.0 software and displayed as the mean ± S.E.M. The statistical significance of the difference was assessed using Student's *t*‐test, and the one‐way ANOVA with Tukey post‐test was conducted for multiple comparisons. A significant difference was considered when the *p*‐value was less than 0.05 and was represented by *P < 0.05, **P < 0.01, and ***P < 0.001.

## Conflict of Interest

The authors declare no conflict of interest.

## Supporting information

Supplementary
