# Supplementary material for: Macrophages‐Triggered Sequential Remodeling of Endothelium‐Interstitial Matrix to Form Pre‐Metastatic Niche in Microfluidic Tumor Microenvironment
Source: Adv Sci (Weinh). 2019 Apr 10;6(11):1900195. doi: 10.1002/advs.201900195 (PMC6548952; doi:10.1002/advs.201900195)
Supplement: Supplementary file 1 — Supplementary [file ADVS-6-1900195-s001.pdf]

Copyright WILEY-VCH Verlag GmbH & Co. KGaA, 69469 Weinheim, Germany, 2019.

ADVANCED SCIENCE
Open Access

# Supporting Information

for *Adv. Sci.,* DOI: 10.1002/advs.201900195

# Macrophages-Triggered Sequential Remodeling of Endothelium-Interstitial Matrix to Form Pre-Metastatic Niche in Microfluidic Tumor Microenvironment

*Hyunho Kim, Hyewon Chung, Jaehoon Kim, Dong-Hee Choi, Yoojin Shin, Yong Guk Kang, Beop-Min Kim, Sang-Uk Seo, Seok Chung,* and Seung Hyeok Seok**

# Supporting information

# Macrophages-triggered sequential remodeling of endothelium-interstitial matrix to form pre-metastatic niche in microfluidic tumor microenvironment

Hyunho Kim,[1†] Hyewon Chung,[2†] Jaehoon Kim,[1] Dong-hee Choi,[1] Yoojin Shin,[3] Yong Guk Kang,[4] Beop-Min Kim,[4] Sang-Uk Seo,[5] Seok Chung,[1,6]* Seung Hyeok Seok[2]*

[†]These authors contributed equally to this work

[1]School of Mechanical Engineering, College of Engineering, Korea University, Seoul, 02841, Republic of Korea

[2]Department of Microbiology and Immunology, and Institute of Endemic Disease, College of Medicine, Seoul National University, Seoul, 03080, Republic of Korea

[3]Department of Mechanical Engineering, Massachusetts Institute of Technology, Cambridge, MA, 02139, USA

[4]Department of Bio-Convergence Engineering, College of Health Science, Korea University

[5]Department of Biomedical Sciences, College of Medicine, Seoul National University

[6]KU-KIST Graduate School of Converging Science and Technology, Korea University

* Correspondence

Seok (Sid) Chung, PhD

E-mail:sidchung@korea.ac.kr

Seung Hyeok Seok, DVM, PhD

E-mail: lamseok@snu.ac.kr

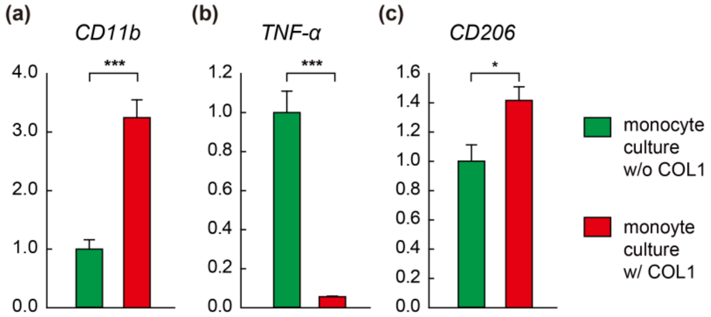


**Figure S1**. Characterization of monocytes within type 1 collagen hydrogel. a-c) mRNA expression levels of markers for macrophage differentiation (M0), M1 macrophage and M2 macrophage in monocytes under 3D culture embedded in type 1 collagen hydrogel. CD11b a), TNF-α, b) and CD206, c) were used as markers for M0, M1 and M2 macrophage respectively. n = 3 independent experiments. Data represent mean ± S.E.M. *, $p<0.05$, ***, $p<0.001$ by Student's t-test

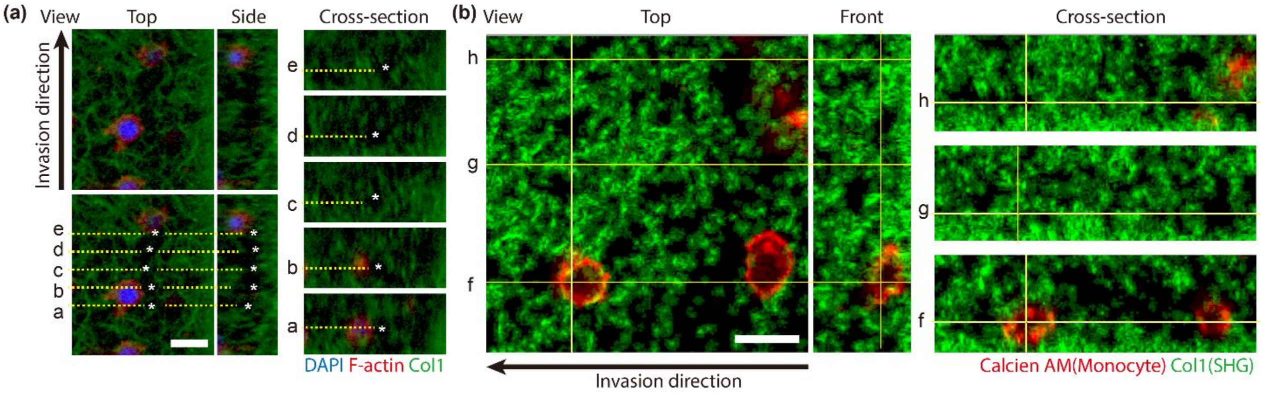


**Figure S2.** Pre-invaded macrophages induced microtracks in collagen. a) Representative confocal fluorescence images of collagen matrix (green), F-actin (red) and DAPI (nucleus, blue) showing collagen structure in the presence of macrophages. a-e indicate position of cross-section view and each asterisk indicates microtracks within the matrix. Scale bar, 20 µm. b) Representative second-harmonic generation (SHG) microscopy images of collagen matrix. f-h indicate position of cross-section view respectively. Scale bar, 20 µm. Calcein AM (red) and collagen fiber (green).

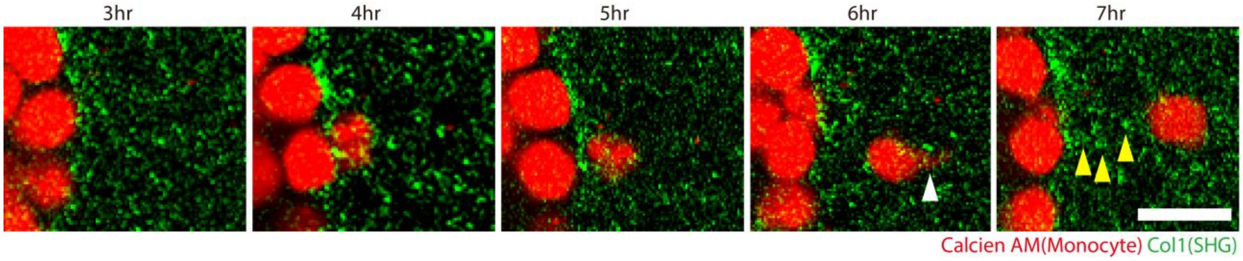


**Figure S3.** Time-lapse SHG microscopy images of migrating monocytes in the collagen matrix (3-7 h after seeding). White arrow head indicates an invadopodia of monocytes and yellow arrow heads indicate deformation of collagen matrix respectively. Calcein AM (red) and collagen fiber (green). Scale bar,20 μm.

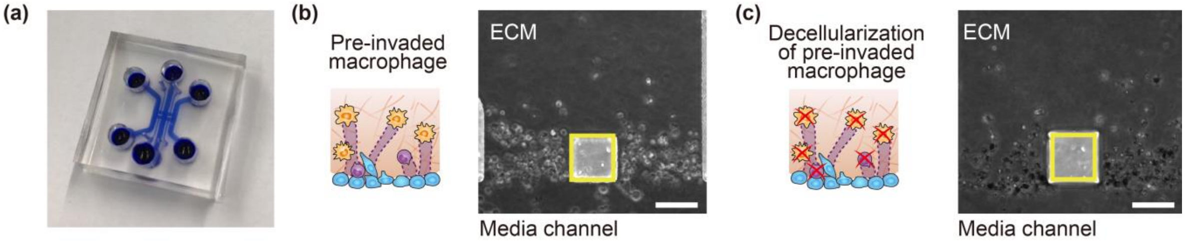


**Figure S4**. Decellularization of pre-invaded macrophages in microfluidic model. a) To confirm the decellularization with DW, trypan-blue stating was performed on a microfluidic chip. b and c) Illustration of the experiment (left panel) and representative phase images (right panel) of pre-invaded macrophage without (b) or with decellularization process (c). Dead cells were stained with trypan-blue dye. Scale bar, 150 μm.
